# Report of Cases

**Published:** 1902-06

**Authors:** R. R. Kime

**Affiliations:** Atlanta, Ga.


### REPORT OF CASES.*

CASE 1. TRANSVERSE RUPTURE PERINEUM. CASE 2. EMBOLISM DURING LABOR. CASE 3. FILARIA SANGUINIS HOMINIS, COMPLICATING PREGNANCY AND LABOR.

BY R. R. KIME, M.D.,
ATLANTA, GA.

CASE 1. Mrs. S. Primipara, age twenty-three, married two years. Pregnancy normal, no albumin, no casts in urine. Confined January 14, 1902.

First stage of labor normal, lasting about eight or nine hours; second stage progressed very well except perineum somewhat rigid, but was yielding under hot applications and cautious use of chloroform.

Feeling something unusual through the napkin (cloth wrung out of a hot lysol solution), I exposed parts to view and found a transverse laceration of perineum at outlet of bowel. The rent extended transversely across into skin surface one to two inches to each side of rectum. The remaining part of the perineum was spread out over presenting part of child's head with nose and mouth of fetus presenting through the rent and vertex through vulva.

I immediately divided the perineum through median line with scissors and allowed the child's head to escape. After delivery brought parts together with catgut and silk sutures, placing two sutures transversely in skin surface on each side of median line of perineum.

*Read before the Georgia Medical Association, Savannah, April, 1902.

Having a nurse that did not recognize the necessity of asepsis, the parts became infected about the fifth day and part of the stitches had to be removed, all at the end of the first week. The corners of the skin flaps sloughed off, but a bridge of tissue at the fourchette held parts together.

The uterus became infected, developing left pelvic phlebitis, which gradually cleared up by irrigation and drainage of uterus and constitutional treatment. Notwithstanding the irrigation and drainage of womb we secured very good union of parts, and patient will get along very well without secondary operation on the perineum.

CASE 2. Mrs. M., age twenty-four, second pregnancy, having miscarried about the third month in previous pregnancy. Patient at full term: no albumin nor casts in urine, her general condition good except a slight cold, with some tendency to cough.

At commencement of labor excess of liquor amnii was noted with low implantation of placenta, margin being felt with examining finger, but no special hemorrhage.

Also noticed bulging of uterine wall a little larger than a fetal head, central portion elevated about two inches above general contour of womb, and situated to right of median line about midway between cervix and fundus, not a solid but fluctuating elevation.

Labor otherwise normal until completion of second stage, with some laceration of perineum.

After delivery of child, severing cord before any special effort at delivery of placenta was made, patient grew suddenly pale and pulseless without special hemorrhage; at first thought she was simply fainting, but soon saw she was gasping for breath and we had something more serious to deal with. Patient had but little anesthetic and no pillow under head. Immediately gave strych. 1-60, nitroglycerine 1-100, digitalis 1-100 gr. hypodermically at 5 A.M.; 6:10 A.M. gave strychnine 1-30, nitroglycerine 1-100 gr., followed with salt solution under left breast. At 6:35 gave strych. 1-60, dig. 1-100 gr., followed with salt solution under right breast, in all about two pints.

Aromatic spts. am. was also given at intervals; at 8 A. M. strych. 1-60, dig. 1-100 gr.

Most of the time patient was almost pulseless, unable to count them, at times gasping for breath, all the while fully conscious.

At 10:10 A.M., pulse 140 to 160.

At 10:30 A.M., pulse 130 to 140; digitalis 1-100 gr.

At 12:15, pulse 140; 12:30, strych. 1-60, dig. 1-100.

At 1:30 P.M., pulse 130.

At 2:00 P.M., pulse 120.

Placenta was delivered about 8:30 A.M. without special difficulty.

In about two hours patient had received 1-12 gr. strych., 1-33 gr. digitalis, 1-50 gr. nitrogly., 2 pints salt solution and about 1 tablespoon aromat. spts. ammonia.

The next day pulse ranged 110 to 130 per minute. Temperature 100 to 101° F. each, gradually subsiding, reaching normal in about two weeks.

Very slight cough; no pneumonic inflammation perceptible.

We are at a loss to decide in this case as to character and location of embolism. Was it air embolism from uterus, a giant placental cell (syncytium), or a blood-clot from uterine sinuses?

Was it pulmonary or cardiac in location, and what the cause?

It was not septic in character, no air or fluid had been thrown into uterine cavity.

CASE 3. Mrs. F., age thirty-five. First consulted me in May, 1901, for hematuria, with which she had been suffering for several weeks.

Examination of urine revealed the presence of blood and chyle. The patient gave the following history: Born in Charleston, S. C., residing there until twenty-two years of age; then in Macon, Ga., one year, 1890; next in Alabama two and one-half months; then in Charleston nine years until moving to Atlanta. Had an attack of hemorrhage from kidneys at the age of seventeen years, April to October.

Second attack at twenty to twenty-one years, lasting four months.

While in Macon had malaria seven months, chills and fever, with an abscess of considerable size on right arm. Blood not examined at that time. Dr. Simmons of South Carolina found the filaria in the blood at 8 P.M. when patient was twenty-two years of age, some time after second attack of hematuria. Soon after patient consulted me I had two spells of sickness myself and left the city for two weeks. Drs. Cromer and Collins examined blood at various times—8 P.M., 10 P.M. and early morning hour—but failed to find filaria.

Patient improved, urine cleared up under treatment, but on examination of patient in July decided she was pregnant. In November hematuria returned, blood examined at 5 p.m. by Dr. Collins and myself and filaria found.

Under treatment patient improved and urine cleared up.

Trouble returned again in February, urine containing blood and chyle, no casts, with heat and nitric acid deposited, albumin about one-half by volume. As blood cleared up the chyle seemed to increase for awhile, urine white color, very thick, gelatinous and unable to pour it from bottle until it was broken up with a glass rod. Filtered specimen contained about same proportion of albumin. Patient frequently had difficulty in passing urine, due to the thick, gelatinous condition. Urine had almost returned to normal condition at confinement, March 15, 1902. Labor, first stage normal, second stage very short, third stage normal. Temperature after labor did not exceed 100 F. almost constantly, 99 in afternoons, continuing up to April 19, one month after labor.

Ten days after labor no filaria found in the blood, child's blood not examined. At no time were filaria found in the urine. Capsules containing thymol, salol and boric acid were found most efficient in controlling the hematuria and chyluria.

Mild diuretics and Lithia water were given, also tonic containing strychnia and colored fluid hydrastis at mealtime. Ergot could not be given on account of pregnancy.

REMARKS.

The most common varieties of the filaria sanguinis hominis are nocturna, diurna and perstans, occurring as their names indicate. They are found in tropical and temperate climates, said to occur in Virginia, Alabama, South Carolina and Florida. Most cases reported are from South Carolina. The mosquito is considered the intermediary host, conveying the ovum to drinking-water. By this means the probabilities are that an increased number of cases will occur in the United States. No specific treatment has yet been found. The most common results of malaria are hematuria, chyluria, elephantiasis and lymph scrotum.

